# The autophagy process

**DOI:** 10.18632/oncotarget.15951

**Published:** 2017-03-07

**Authors:** Sushil Devkota

**Affiliations:** Section of Cell and Developmental Biology, University of California, San Diego, La Jolla, CA, USA

**Keywords:** autophagy, lysosome, molecular-signaling, protein-degradation

A day in the life of a cell,
There are so many stories to tell.

Ribosomes are cooking proteins,
It smells delicious in cells kitchen;
The beauty of the genetic code,
Is inside the Nucleus laid hidden.

Kinases are busy kissing proteins,
They leave the lip mark of phosphate on them;
Phosphatases are running here and there with jealousy,
Removing phosphate is their game.

Of all these stories inside a cell,
Those are required to keep the cell neat;
This is the story of autophagy,
The process by which cells ‘self-eat’.

When there is dark everywhere,
And not enough food on the table;
When cells feel unstable,
And bacteria, viruses, and oncogenes prevail.

A glimmer of hope emanates in the cytosol,
Wearing the double membranous veil;
‘So do not fear’ Autophagy roars,
‘I declare war against adversities and will prevail’.

Collecting intelligence cues from diverse signaling,
Autophagy breaks shackles of mTOR;
ULKs and Class III PI3K complex next join the war,
The first battalion is formed at the phagophore.

Mimicking the Ubiquitin system,
ATG5-ATG12-ATG16 then join the noble cause;
This war is about ‘to be or not to be’,
There is no excuse for rest or a pause.

Final reinforcement of LC3,
Ultimately decorates the lipid bilayer;
The membrane is going to form a circle,
Engulfed materials will soon disappear.

Nevertheless, like in any war,
Autophagy complains-‘Rumors were spread against me’;
‘Autophagy is brutal’-it was told,
Engulfs and kills targets indiscriminately’.

Alas, they did not know,
Autophagy can be very selective;
Autophagy receptors p62 or NBR1,
Perform the selective autophagy trick.

Mitophagy for mitochondria, lipophagy for lipids,
And pexophagy for the peroxisomes;
There are many secrets of autophagy hidden,
Those are yet to be known.

Autophagosome dumps the cargo on the lysosome,
An organelle full of protease knife;
All the contents are then chopped to be recycled,
Executing the beautiful ‘art-of-life’.

This is the story of Autophagy,
Keeping the cell clean its ultimate motive;
Autophagosome ends its life in the lysosome,
But the cell continues to live.

